# Maladaptive trained immunity in viral infections

**DOI:** 10.1172/JCI192469

**Published:** 2025-09-02

**Authors:** Dmitri Sviridov, Mihai G. Netea, Michael I. Bukrinsky

**Affiliations:** 1 Baker Heart and Diabetes Institute, Melbourne, Victoria, Australia.; 2 Department of Biochemistry and Molecular Biology, Monash University, Clayton, Victoria, Australia.; 3 Department of Internal Medicine and Radboud Center for Infectious Diseases, Radboud University Medical Center, Nijmegen, Netherlands.; 4 Department of Immunology and Metabolism, Life and Medical Sciences Institute, University of Bonn, Bonn, Germany.; 5 Department of Microbiology, Immunology, and Tropical Medicine, The George Washington University School of Medicine and Health Sciences, Washington, DC, USA.

## Abstract

Trained immunity (TRIM) is a form of long-lasting functional reprogramming of innate immune cells and their progenitors that enhances responsiveness to subsequent stimuli. Although first characterized in myeloid cells, TRIM was recently extended to nonmyeloid cell types, including endothelial and glial cells, which also exhibit stimulus-driven, memory-like behavior. While initially recognized as a protective mechanism, particularly in the context of vaccines and acute infections, TRIM can also become maladaptive, promoting chronic inflammation, immune dysfunction, and disease. This Review focuses on virus-induced TRIM while also addressing microbial, metabolic, and endogenous inducers. We examine key ligands and receptors that initiate TRIM and dissect the associated signaling and epigenetic pathways. Importantly, we argue that maladaptive TRIM arises not from a specific ligand, receptor, or molecular event, but from contextual factors such as stimulus persistence, dose, tissue microenvironment, and preexisting inflammation. The nature of the secondary challenge also shapes whether a trained response is adaptive or maladaptive. We further discuss TRIM induction in the bone marrow, involvement of both myeloid and nonmyeloid cells, and the role of lipid rafts in sustaining TRIM. We review maladaptive TRIM’s potential contribution to systemic diseases, such as atherosclerosis, diabetes, sepsis, cancer, and autoimmunity, along with its influence on viral vaccine responses. Finally, we outline potential strategies to redirect maladaptive TRIM and propose key outstanding questions for future research.

## Introduction

**Figure 1:**
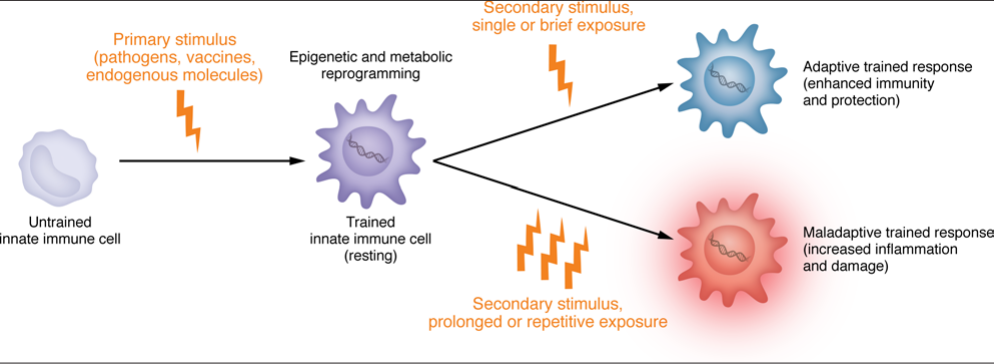
The spectrum of TRIM responses: from protection to pathology. TRIM is initiated by microbial, vaccine-derived, or endogenous stimuli that trigger epigenetic and metabolic reprogramming of innate immune cells. The outcome of TRIM depends on the nature and duration of exposure. Single or brief exposures typically promote *adaptive TRIM*, enhancing immunity and protection. In contrast, prolonged or repetitive stimulation, such as that occurring in chronic infections such as HIV, can lead to *maladaptive TRIM*, characterized by persistent inflammatory responses that contribute to tissue damage and disease. This conceptual framework helps explain both beneficial and detrimental consequences of trained immunity across different contexts.

The notion of TRIM was introduced to describe the capacity of innate immune cells to acquire memory-like properties following exposure to specific stimuli, resulting in enhanced responses to subsequent infections, including those caused by unrelated pathogens (1). Originally proposed to explain the nonspecific benefits of live vaccines such as Bacillus Calmette–Guérin (BCG), TRIM has since been linked to a wide range of protective and pathological outcomes. Its mechanistic basis lies in epigenetic and metabolic reprogramming of cells (2). While initially observed in monocyte-lineage cells, the concept of TRIM has expanded to include nonmyeloid populations such as endothelial, epithelial, and smooth muscle cells, which also display stimulus-driven, memory-like functional reprogramming (3–5). While TRIM is a beneficial adaptation in acute infection and vaccination contexts, persistent or dysregulated activation may lead to maladaptive outcomes, contributing to chronic inflammation and disease. To describe this phenomenon, the concept of maladaptive TRIM was introduced (6). The dual nature of TRIM is increasingly recognized as a context-dependent phenomenon. While transient or controlled exposures, such as vaccination or acute infection, can induce adaptive TRIM that bolsters host defense, chronic or repeated stimuli may drive maladaptive TRIM, sustaining inflammation and contributing to pathogenesis (Figure 1). This Review explores the molecular basis of TRIM and its divergent outcomes in health and disease — emphasizing maladaptive TRIM in the setting of viral infections such as HIV — with the goal of clarifying its mechanisms, consequences, and therapeutic implications. A deeper understanding of maladaptive TRIM is critical for identifying new strategies to mitigate inflammatory comorbidities and long-term complications associated with chronic or recurrent viral exposure.

## Viral infections and TRIM

Innate antiviral immune responses are a critical component of host defense and play a central role in determining the outcome of viral infections; many viral infections induce features of TRIM (7). TRIM has been documented following infections with a variety of viruses, including HIV (8–10), SARS-CoV-2 (11, 12), hepatitis B virus (HBV) (13), enteroviruses (14), respiratory syncytial virus (RSV) (15), and white spot syndrome virus (WSSV) (16). Consistent with these findings, several live attenuated viral vaccines have been associated with nonspecific protective effects, as evidenced by a decreased incidence of infections unrelated to the vaccine target and reduced all-cause mortality. Such effects have been reported following vaccination with measles (17), smallpox (18), and oral poliomyelitis vaccines (19). Live attenuated influenza vaccine has also shown cross-protection against subsequent SARS-CoV-2 infection in humans (20) and RSV in mice (21). Similarly, infection with SARS-CoV-2 was shown to protect mice from lethal influenza challenge (22). Notably, the ability of the virus or vaccine to replicate appears to be a key requirement for inducing long-lasting TRIM, as inactivated influenza vaccines failed to confer cross-protection against RSV in murine models (21).

In contrast to the adaptive (protective) TRIM induced by live attenuated viral vaccines, chronic viral infections, such as HIV (23), human cytomegalovirus (HCMV) (24), and chikungunya virus (25), are frequently associated with sustained inflammation, a hallmark of maladaptive TRIM. These seemingly contradictory outcomes raise a fundamental question: What determines whether TRIM manifests as a protective or pathological response?

One proposed resolution to this paradox is that TRIM is initially established upon the first exposure to a viral pathogen or vaccine, potentially even in its inactivated form, but may be subsequently reshaped by ongoing stimulation. In vitro studies have demonstrated that a single exposure to a TRIM-inducing agent can establish long-lasting reprogramming in monocytes without requiring persistent activation (26). However, prolonged or repetitive stimulation may alter this trajectory. Depending on the duration, frequency, and intensity of exposure, continuous immune activation may either downregulate inflammatory responses to promote protection or perpetuate inflammation and contribute to maladaptive TRIM. The former scenario is exemplified by live attenuated vaccines, which typically replicate for a limited period, up to a few weeks, before being cleared by the host immune system (27–29). In contrast, persistent immune stimulation is seen in chronic HIV infection, where extracellular vesicles (EVs) carrying the viral protein Nef continuously activate innate immune cells and sustain a proinflammatory TRIM phenotype (9). Similarly, the intensity of immune activation appears to influence TRIM outcomes: for instance, SARS-CoV-2 infection in humans is often associated with a cytokine storm and systemic inflammation, potentially predisposing to maladaptive TRIM. In contrast, SARS-CoV-2 infection in mice, which lacks such exaggerated inflammation, has been associated with protective TRIM (22). Cytokine storm may also lead to immune exhaustion or tolerance — an alternative maladaptive immune state associated with impaired responses. Furthermore, repeated infections and/or vaccinations with SARS-CoV-2 may contribute to the development of maladaptive TRIM and may play a role in the pathogenesis of long COVID (30).

The following sections explore the cellular and molecular mechanisms underlying maladaptive TRIM in greater detail.

## Maladaptive TRIM: concept and mechanisms

Emerging evidence implicates TRIM in the pathophysiology of chronic inflammatory diseases, including atherosclerosis, sarcoidosis, Crohn’s disease, and gout (31–34). The concept of maladaptive TRIM has been introduced to describe situations where an initial infection or inflammatory condition, such as periodontitis or rheumatoid arthritis (6, 35), induces trained immunity in bone marrow myeloid progenitor cells, thereby exacerbating and prolonging inflammatory responses during current or subsequent infections. Ultimately, maladaptive TRIM may contribute to, and even be a major cause of, chronic inflammation after the initial insult has been controlled or eliminated, increasing the risk of comorbidities or inflammatory sequelae. IL-1β signaling has been identified as a critical mediator in this process (6). However, several key questions must be addressed to transition this concept from a theoretical phenomenon to a practically applicable framework: (i) why TRIM is protective in some cases but maladaptive in others; (ii) what factors determine the trajectory of TRIM; and (iii) when, how, and where these decisions are made. Answering these questions requires a comprehensive mechanistic understanding of the molecular pathways governing the establishment and modulation of TRIM. This understanding has only recently started to emerge. Here, we provide an overview of relevant publications and proposed mechanisms that might explain the development of maladaptive TRIM.

## TRIM inducers and engaged receptors

The nature of the TRIM-inducing agent and its receptor may influence outcomes of training. Complex microbial structures, such as viruses or BCG, engage several receptors. It is challenging to associate a particular component with a specific TRIM program (maladaptive TRIM; protective [adaptive] TRIM; or dual-action TRIM, referring to a component that may induce either a maladaptive or protective response). Similarly, endogenous ligands such as oxLDL (36) and Lp(a) (37) exert a combination of multiple effects, including those of oxidized proteins (e.g., apoB), oxidized phospholipids (37), and induced cellular metabolites, such as accumulation of cholesterol (38). However, TRIM can also be induced by simpler and well-defined structures. Polysaccharides such as β-glucan (associated predominantly with protective TRIM) (39) and LPS (known to initiate dual action TRIM) (40) are potent inducers of TRIM. Proteins like HIV Nef (linked to maladaptive TRIM) (9) and even short peptides such as DCATH-2, an analog of the host defense peptide cathelicidin-2 composed of 26 D-amino acids, have been shown to induce TRIM (41). Various lipids can also act as adjuvants or independent TRIM inducers (42), including LDL-cholesterol (38), phospholipids (37), saturated fatty acids (43, 44), and aldosterone (45). Catecholamines (45, 46) and nucleic acids, such as RNA in mRNA vaccines (47), have also been identified as TRIM inducers. The list of TRIM-inducing compounds is rapidly expanding. There is little evidence, however, that the chemical structure of the inducer unequivocally determines the subsequent type of the response, maladaptive or protective, and association of some with a particular type of TRIM is most likely due to the experimental system used to characterize the effect of that particular agent.

Different stimulating agents interact with distinct receptors, offering another potential explanation for variations in long-term phenotypes. Both cell-surface and intracellular receptors have been implicated in the induction of TRIM. For instance, β-glucan interacts with the surface receptor dectin-1 (48), while fatty acids bind to TLR4 (43), which is also used by LPS (49). Intracellular receptors also play a role: one of the receptors engaged by BCG is NOD2 (50), while aldosterone engages the mineralocorticoid receptor (45). Lp(a) can activate monocytes through TLR2 and NF-κB signaling (51). The induction of TRIM by DCATH-2 involves the purinergic receptor P2X7R, which reacts with endogenous ligands. Notably, DCATH-2 must first undergo internalization via a P2X7R- and lipid raft–dependent pathway (41). Some stimulators bypass classical receptor-binding mechanisms altogether, instead targeting enzymes or metabolic pathways. For example, while HIV Nef can induce maladaptive TRIM (9), there is no evidence that the Nef protein itself binds to or signals through a specific cell surface receptor. Rather, the biological effects of Nef carried by EVs are attributed to its intracellular delivery, either through direct uptake of EVs or endocytosis, followed by interactions with intracellular host proteins involved in signaling, trafficking, and transcriptional regulation (52).

Certain agents require both a receptor and metabolic perturbation. For instance, oleic acid induces TRIM through TLR4 and ceramide biosynthesis (44), while aldosterone requires its receptor and active fatty acid synthesis (45). In contrast, palmitic acid causes maladaptive TRIM, but the addition of oleic acid blocks this effect by competing for ceramide biosynthesis (44).

Plant polyphenols, such as resveratrol, can modulate TRIM outcomes. Resveratrol enhances BCG-induced protective TRIM and inhibits maladaptive TRIM formation induced by oxLDL (53). Interestingly, oxLDL requires LXRα (but not LXRβ) activity for TRIM induction, with LXRα agonists further potentiating this effect (54). While LXRα regulates pathways linked to inflammation and trained immunity, the precise connection between oxLDL and the LXRα network remains unclear, aside from oxLDL’s role in macrophage loading with cholesterol and oxysterols; specifically, some oxysterols may act as LXR agonists (55). The outcome of trained immunity induced by one stimulus can also be modified by another, as exemplified by β-glucan reversing LPS-induced tolerance, a process largely mediated through suppression of IL-10 signaling (56). Table 1 summarizes the knowledge discussed above, listing TRIM inducers, their corresponding receptors, and known modulators.

In addition to immunological effects, TRIM induction is accompanied by changes in cellular metabolism of innate immune cells. Despite the diversity in chemical structure and initiating mechanisms, the downstream metabolic effects leading to TRIM of both types remain remarkably similar: activation of aerobic glycolysis, cholesterol biosynthesis, and fatty acid synthesis, leading to epigenetic modifications (57). Moreover, studies on maladaptive TRIM in patients with granulomatosis identified pathways such as mTOR signaling, cholesterol biosynthesis, and glycolysis that are also central to protective TRIM (32, 58). Ultimately, the manifestation of TRIM, whether adaptive or maladaptive, appears not to be determined by the chemical nature of the trigger or the identity of the receptor or the metabolic pathway it engages.

## Bone marrow involvement

An important observation that needed to be explained was the long-term persistence of TRIM effects, which occur despite a short (less than a week) half-life of innate immune cells in the circulation (59). Thus, it has been described that TRIM programs are also induced in the progenitor innate immune cell populations in the bone marrow (60). When these precursors are engaged, they ensure persistent production of cells with a TRIM phenotype, increasing the likelihood of persistent inflammatory responses, as demonstrated in TRIM induced by Nef-carrying EVs (9), stroke (33), myocardial infarction (61), enterovirus (14), elevated plasma levels of cholesterol (38, 62), and glucose (63). In contrast, when bone marrow progenitors are not affected, TRIM responses are relatively short-lived and manifest as a protective mechanism devoid of persistent inflammatory response, as observed in SARS-CoV-2 infection in mice (22). However, such a contrasting difference between central and peripheral innate memory is rare, and there are many examples, including the classical BCG-induced TRIM, where TRIM formed in bone marrow hematopoietic progenitor cells leads to the formation of a protective phenotype (64). Most often, TRIM-inducing agents involve both peripheral and central types of innate memory, and reasons for maladaptive TRIM may be more nuanced.

The engagement of specific subpopulations of very early hematopoietic stem and progenitor cells (HSPCs) may influence the specificity of TRIM responses and, consequently, their maladaptive potential. HSPCs represent a heterogeneous group, including long-term hematopoietic stem cells (LT-HSCs) and short-term hematopoietic stem cells (ST-HSCs), which differ in their self-renewal capacities (65). Different TRIM agents may selectively target specific HSPC subsets or their subpopulations (66), leading to different outcomes of TRIM. However, evidence suggests that activation of both HSPC types can result in favorable TRIM outcomes. For instance, LT-HSC activation by agents such as BCG and β-glucans has been shown to induce protective TRIM (64, 67, 68). Similarly, ST-HSC activation by heme also promotes advantageous TRIM effects (69).

## Engagement of nonmyeloid cell types

TRIM was initially proposed as a property of the innate immune system, primarily associated with monocytes/macrophages and their bone marrow progenitors. However, metabolic and epigenetic reprogramming events analogous to those observed in macrophage-mediated TRIM have also been documented in other cell types, including endothelial cells (70), microglia (71), astrocytes (72), splenocytes (73), and epithelial cells (74). These findings raise the possibility that TRIM-like mechanisms in nonmyeloid cells may influence the trajectory toward either adaptive or maladaptive inflammatory states (57). For instance, the progression of atherosclerosis is accelerated when inflammatory activation occurs simultaneously in both macrophages and endothelial cells (75, 76). Similarly, neurodegeneration advances more rapidly when microglia-driven neuroinflammation coincides with blood-brain barrier disruption due to endothelial damage (77). This convergence of TRIM-induced inflammatory amplification across multiple cell types and pathways may critically shape the inflammatory microenvironment, favoring maladaptive outcomes.

## Quantitative and qualitative factors

The balance between adaptive (protective) and maladaptive forms of TRIM is likely shaped by both quantitative and qualitative factors related to the induction stimulus and the broader disease context. Variables such as preexisting inflammation unrelated to TRIM and the dose, duration, frequency, and intensity of the TRIM-inducing exposure can shift the immune response toward either protective or pathological outcomes (71). This hypothesis is supported by observations in chronic viral infections, notably HIV, where persistent inflammatory comorbidities correlate with prolonged exposure to TRIM-inducing agents such as the viral protein Nef. Repeated or chronic stimulation may drive durable epigenetic reprogramming that favors maladaptive TRIM (77, 78).

Importantly, the trajectory of TRIM appears to be context dependent: a short-lived microbial stimulus during an acute, self-limited infection may yield beneficial immune memory and protection against reinfection. In contrast, sustained or repetitive exposure to endogenous or exogenous inducers can lead to dysregulated responses, persistent inflammation, and maladaptive TRIM (Figure 1).

The distinction between adaptive and maladaptive TRIM likely occurs at two levels: first, at the stage of primary TRIM induction, when stimulus characteristics shape the epigenetic landscape; and second, at secondary stimulation, when a new challenge interacts with the trained state. If a secondary infection overwhelms the protective benefits of TRIM and triggers persistent inflammation, the originally adaptive TRIM may enhance this inflammatory response, thus eliciting a maladaptive state. This functional shift is illustrated in the following section, with examples from several chronic diseases.

## Context-dependent consequences of TRIM in disease

### Cardiometabolic diseases.

Cardiometabolic diseases such as atherosclerosis and diabetes offer an instructive context to examine how TRIM may evolve into either an adaptive or maladaptive state. Both diseases feature chronic inflammation as a key component of their pathogenesis and lifelong exposure to abnormally high levels of known inducers of TRIM: cholesterol in atherosclerosis and glucose in diabetes. It is not surprising, therefore, that both atherosclerosis (37, 61, 63) and diabetes (63, 79, 80) have been associated with the development of TRIM.

All available evidence points to high concentrations of glucose as the key inducer of TRIM in diabetes (63, 79, 80). There is, however, limited knowledge about the contribution of TRIM to specific pathogenic processes in diabetes, such as impairment of insulin secretion, insulin resistance, or adipose inflammation, and its impact on diabetes severity. Thus, one report is consistent with the formation of maladaptive TRIM (81), while another suggests the formation of adaptive TRIM (82).

In atherosclerosis, TRIM was shown to be induced by various triggers: high plasma cholesterol (38), oxidized LDL (36, 37, 54, 80), hyperglycemia (63), adrenaline and noradrenaline (45), aldosterone (45), infection (83), and unknown substances released into the systemic circulation after myocardial infarction (61), among others (for review see ref. 84). All these compounds and conditions are capable of inducing TRIM on their own, i.e., they are bona fide inducers of TRIM, not merely modifiers or boosters.

TRIM is formed in various cells involved in the pathogenesis of atherosclerosis, including monocytes/macrophages (36, 45, 54, 63, 80, 85), bone marrow cells (38, 86), and endothelial cells (70). While TRIM was consistently associated with markers of enhanced atherosclerosis, only a few studies demonstrated a direct contribution of TRIM to the progression of atherosclerotic plaque to prove its maladaptive nature (63, 85). Most studies were limited to demonstrating the presence of elements of TRIM in atherosclerosis, without assessing its contribution to vascular inflammation or plaque growth.

In summary, while substantial evidence supports the presence of TRIM in both atherosclerosis and diabetes, its role, whether adaptive or maladaptive, remains incompletely defined. Chronic exposure to potent TRIM inducers such as oxidized LDL, cholesterol crystals, and high glucose levels provides a persistent training environment, but it is the nature of the secondary inflammatory challenge, such as tissue injury, infection, metabolic fluctuation, or acute cardiovascular events, that ultimately reveals the consequences of this trained state. In atherosclerosis, for instance, TRIM may enhance monocyte responsiveness to TLR ligands released during infection or following myocardial infarction, thereby amplifying vascular inflammation or accelerating plaque progression (87). In diabetes, trained monocytes or macrophages may exhibit exaggerated inflammatory responses to adipose tissue stress or islet-derived damage-associated molecular patterns (DAMPs), potentially worsening insulin resistance or β cell dysfunction (88). Yet many studies have stopped short of directly linking trained responses to such downstream pathological events. Conflicting findings, some suggestive of protective immune enhancement, others pointing to pathological amplification, highlight the need to contextualize TRIM within specific immunometabolic and clinical settings. Establishing clear causal links between TRIM and disease progression will be essential to distinguish adaptive from maladaptive TRIM and to inform therapeutic strategies aimed at modulating these responses in cardiometabolic disease.

### Cancer and autoimmunity.

Two other chronic conditions associated with the formation of TRIM are cancer, where the role of epigenetic modifications is firmly established, and autoimmune diseases, where sustained inflammation is the principal driver of pathogenesis. The formation of TRIM in both conditions was discussed in a recent review (89). In cancer, similar to viral infections, TRIM may be both adaptive and maladaptive, on one hand boosting and prolonging the anticancer immune response, but on the other hand enhancing and sustaining inflammation, which in many cases is carcinogenic. While it has not been firmly established what drives the conversion of adaptive into maladaptive TRIM in cancer, it likely depends on the specific context of cancer progression. In contrast, in autoimmune diseases, similar to chronic infections, TRIM is predominantly maladaptive, sustaining persistent inflammation (90–92).

### Sepsis.

Another disease in which TRIM can play a dual role is sepsis (93). In its adaptive form, TRIM enhances pathogen recognition and clearance, promoting early containment of infection. For example, administration of β-glucan or BCG has been shown to induce TRIM that protects against lethal sepsis in mice by boosting early myelopoiesis and cytokine responsiveness (94–98). However, if inflammation is not effectively resolved, this heightened state can shift into a maladaptive form of TRIM. This maladaptive state is characterized by sustained epigenetic activation of inflammatory genes and metabolic pathways, such as glycolysis, resulting in persistent or exaggerated inflammatory responses. Such hyperinflammation is a hallmark of one important immunotype of severe sepsis and is associated with increased risk of organ failure and death (99, 100).

Recent evidence suggests that prior viral infections can induce maladaptive TRIM that worsens outcomes in bacterial sepsis. For example, influenza infection primes monocytes to overrespond to subsequent bacterial pneumonia, leading to fatal lung injury and sepsis-like symptoms (101, 102). Similarly, prior influenza A virus infection epigenetically reprograms innate immune cells, which results in excessive IL-1β and neutrophil responses during secondary *Staphylococcus aureus* infection, amplifying sepsis severity (103). In a striking example of virus-induced maladaptive TRIM, SARS-CoV-2–induced TRIM underlies cytokine storm, multiple organ damage, and mortality, implicating it as a risk factor for severe sepsis (104). These studies highlight how maladaptive TRIM initially triggered by viral infections can dysregulate subsequent innate responses and aggravate sepsis pathogenesis.

### Aging.

Finally, aging is an interesting paradigm for the formation of adaptive versus maladaptive TRIM. On one hand, aging is associated with weakening of the adaptive immune system, increasing the importance of TRIM in overall immune protection. On the other hand, the probability of multiple exposures and underlying chronic inflammatory diseases increases with age. Most available data indicate that TRIM in the elderly is protective and can, at least partially, compensate for age-related immune deficiency (105–107). However, maladaptive TRIM has been implicated in poor prognosis in traumatic brain injury in aged mice (108). Since TRIM formation in aging likely involves multiple inducing factors, the ultimate outcome of a particular disease in aged individuals likely depends on the disease context, consistent with observations in other settings.

## TRIM and lipid rafts

The key metabolic features necessary for the initiation of TRIM were originally identified as a metabolic switch toward oxidative glycolysis and an enhanced rate of cholesterol biosynthesis (109). In our study on TRIM formation in response to HIV infection, we described an additional metabolic feature critical for TRIM induction, specifically in the context of HIV: an increased abundance of lipid rafts (9). This finding was corroborated by observations that pathological inflammorafts — abnormal, more stable, enlarged, and/or clustered lipid rafts — persist in non-foamy macrophages within atherosclerotic lesions and promote macrophage reprogramming into a hyperinflammatory phenotype (85).

The role of lipid rafts in TRIM formation is further supported by the proposed critical involvement of insulin-like growth factor 1 receptor (IGF1R), a lipid raft–associated protein (109). The activity of receptors located in lipid rafts is often regulated by changes in raft abundance and properties. Many pattern recognition receptors, including TLRs, along with their associated signaling machinery, are also localized in lipid rafts. Their activity is critically dependent on this localization, and thus on raft abundance (110). Formation of TRIM in response to aldosterone was shown to depend on fatty acid synthesis and specifically on the activity of fatty acid synthase (FASN) (45). FASN influences cholesterol biosynthesis and lipid raft integrity (111, 112). Additionally, lipid rafts house elements of several endocytic pathways essential for TRIM inducers with intracellular targets. For instance, TRIM formation in response to cathelicidin-2 depends on caveola/lipid raft–mediated uptake of the peptide (41). The enhanced rate of cholesterol biosynthesis associated with TRIM not only increases concentrations of mevalonate and acetyl-CoA, previously proposed as key mechanistic elements in TRIM formation (109), but also elevates lipid raft abundance (113). Notably, many genes encoding raft-associated components undergo epigenetic regulation, akin to that observed in TRIM (114).

It remains unclear whether lipid raft involvement is characteristic of all instances of TRIM formation or only specific cases. Additionally, it is unknown whether lipid rafts favor protective or maladaptive TRIM. However, studies directly examining lipid rafts have primarily focused on maladaptive phenotypes (9, 85). If lipid rafts are indeed selectively implicated in maladaptive TRIM and contribute to persistent low-grade inflammation, this opens the possibility of leveraging “lipid raft therapy” (115) to mitigate inflammation and address the metabolic and neurological comorbidities associated with viral infections.

## Tissue-resident TRIM

Tissue-resident TRIM expands the classical concept of innate immune memory by incorporating the contribution of monocyte-derived macrophages that persist and adapt within tissues following infection or inflammation. While traditional TRIM is attributed to epigenetic reprogramming of bone marrow progenitors or circulating monocytes, recent studies have demonstrated that monocyte-derived tissue macrophages can acquire long-lasting memory-like traits even in the absence of canonical histone modifications. This phenomenon is particularly well documented in the lung, where respiratory infections such as influenza destroy the natural pool of yolk sac–derived alveolar macrophages and drive their replacement with monocyte-derived alveolar macrophages (Mo-AMs) (116) that display heightened responsiveness upon reexposure to pathogens (117, 118). Moreover, recent work suggests that noncanonical epigenetic mechanisms, including DNA methylation, long noncoding RNAs, and changes in 3D chromatin architecture, may sustain these altered states even when classical histone marks (e.g., H3K4me3, H3K27ac) are absent (119). These mechanisms may help explain the observed discordances between macrophage phenotype and traditional epigenetic signatures in chronic inflammatory diseases.

In adaptive contexts, this memory-like state is protective. Mo-AMs can enhance local pathogen clearance and amplify early inflammatory signaling, supporting rapid containment of secondary infections. These beneficial effects are often shaped by tissue-derived cues — cytokines, DAMPs, metabolic signals — that instruct the differentiation and imprinting of incoming monocytes. As these cells adapt to the tissue environment, they may acquire semi-stable transcriptional programs that persist over time without the need for classical epigenetic remodeling (22, 120).

However, under persistent or dysregulated inflammatory conditions, these same mechanisms can promote maladaptive TRIM, in which Mo-AMs adopt chronically activated or proinflammatory phenotypes that do not resolve appropriately (121–123). Rather than aiding in host defense, these macrophages contribute to tissue damage, fibrosis, or immune exhaustion. For instance, in models of chronic viral infection or repeated allergen exposure, Mo-AMs have been shown to sustain inflammation well beyond clearance of the initial insult (124). This persistent activation may be driven by continuous NF-κB or mTOR signaling, altered metabolic programming (e.g., aerobic glycolysis), or ongoing exposure to dysregulated tissue cues. As a result, macrophage functions become skewed toward excessive cytokine production and impaired antiinflammatory or reparative responses (125).

Reconciling maladaptive TRIM with the dual capacity of monocytes to mediate both pro- and antiinflammatory responses requires viewing monocyte function as plastic and highly context dependent (126, 127). Under physiological conditions, monocytes contribute to host defense by producing inflammatory cytokines during acute infection and subsequently transitioning to regulatory or reparative phenotypes to resolve inflammation (128). TRIM alters this dynamic by priming monocytes for enhanced responsiveness to secondary stimuli, but the outcome of this process, adaptive or maladaptive, depends on whether this reprogramming remains balanced (129). In cases where the initial training signal is transient and tightly regulated, monocytes respond in most cases more efficiently to reinfection, without disrupting homeostasis. However, chronic or excessive training signals, such as persistent viral antigens or metabolic stressors, may push monocytes into a pathologically sustained proinflammatory state, disrupting their ability to engage in resolution or tissue repair (26, 64). This shift skews monocyte function toward maladaptive TRIM, characterized by exaggerated cytokine production, resistance to regulatory cues, and impaired return to a quiescent or antiinflammatory state (130). Thus, maladaptive TRIM in monocytes reflects a failure of functional flexibility, wherein persistent epigenetic and metabolic priming locks cells into an inflammatory trajectory incompatible with immune resolution and tissue homeostasis.

## TRIM and the shaping of adaptive immune responses

That innate immunity plays a role in guiding adaptive immune responses is a foundational principle in immunology. Antigen-presenting cells such as monocytes, macrophages, and dendritic cells shape T and B cell responses through cytokine production, antigen presentation, and costimulatory signaling. TRIM builds on this classical framework by highlighting how persistent functional reprogramming of innate immune cells, particularly through epigenetic and metabolic remodeling, can alter their long-term capacity to influence adaptive immunity. In this context, it has been shown that the interaction between trained innate immune responses and adaptive immune memory is crucial for effective protection against infections. On the one hand, it has been demonstrated that TRIM-induced type 2 interferon induced by BCG amplifies antiviral responses against SARS-CoV-2 (131). On the other hand, lymphocyte-derived IFN-γ also plays a key role in amplifying trained immunity responses (132–134), suggesting the existence of a positive feedback loop that enhances protection against infections (135). While this trained state can enhance host defense under normal immune responses, accumulating evidence suggests that maladaptive TRIM may distort adaptive immune responses in some individuals, contributing to immunopathology in chronic infections, autoimmunity, and aging (136).

Upon restimulation, trained innate immune cells produce higher amounts of inflammatory cytokines such as IL-1β, IL-6, and TNF-α, which in turn bias T cell differentiation toward Th1 or Th17 phenotypes and sustain effector responses at the expense of regulatory or memory subsets (137). This skewing is beneficial in the context of acute infection or vaccination but can become detrimental in persistent inflammatory states. For instance, monocyte reprogramming induced by chronic viral antigens or metabolic ligands (e.g., oxLDL) can support ongoing low-grade inflammation known to drive T cell exhaustion (138) or disruption of tissue-resident memory T cell homeostasis (139). Similarly, TRIM-altered dendritic cells may improperly amplify B cell activation and antibody production through enhanced costimulatory signals (e.g., increased CD86) and cytokine secretion — phenomena observed in trained DCs from cholera toxin B–induced models (140).

Recent studies demonstrate that BCG-induced TRIM can modulate not only the magnitude but also the specificity and hierarchy of adaptive T cell responses (141). While promising for vaccine enhancement, such broad remodeling raises concerns in disease settings where immune misfiring may have long-term consequences. In HIV infection, for example, persistent exposure to viral proteins and inflammatory EVs may reprogram monocytes in a way that sustains maladaptive inflammation and dysregulates T and B cell responses (142), contributing to impaired immune recovery despite antiretroviral therapy.

Thus, TRIM in its maladaptive form may subvert the normal regulatory balance between innate and adaptive arms, promoting chronic activation, immune deviation, and exhaustion of T cells. Recognizing this duality is essential for designing interventions that harness the protective aspects of TRIM while preventing its pathogenic consequences.

## TRIM and vaccines against viral infections

The broad, nonspecific protection conferred by certain live attenuated vaccines suggests that TRIM can be leveraged to enhance vaccine efficacy against both infectious diseases and cancer (143). This concept has been extensively explored in multiple reviews (144–147). As described earlier, TRIM enhances innate immune responsiveness, which in turn facilitates more rapid and robust activation of adaptive immune responses, including B and T cell immunity. A key determinant of TRIM’s utility is its duration. While vaccines such as those against poxvirus, measles, and poliovirus can confer long-lasting or even lifelong protection, the innate memory induced by BCG is typically shorter lived, often lasting less than a year (148). Although prolonged TRIM may offer sustained protection, it may also increase the risk of inflammatory comorbidities. Ideally, a protective TRIM response should result in short-term heightened responsiveness upon infection, not continuous release of proinflammatory mediators. Nevertheless, certain cytokines, particularly IL-1β, are essential for the protective effects of TRIM, including its roles in antibacterial and anticancer immunity (149). Importantly, not all TRIM responses are proinflammatory. In the context of viral infections, it may be beneficial to steer TRIM toward antiviral pathways, such as the induction of type I interferons and restriction factors, rather than toward excessive production of inflammatory cytokines. Such antiviral TRIM profiles have been observed following AS03-adjuvanted influenza vaccination (150) and in TRIM programs induced by novel COVID-19 vaccines (47). For example, SARS-CoV-2 infection in mice was shown to induce such response that protected against lethal secondary influenza infection by mitigating excessive inflammation (22), though the longevity of this innate memory remains unknown.

While much of the early evidence for TRIM came from in vitro or animal studies, recent human data support the relevance of TRIM in vaccine responsiveness. Wimmers et al. (151) demonstrated that mRNA-based SARS-CoV-2 vaccination reprograms innate immune cells in humans. Their study showed increased chromatin accessibility in monocytes and innate lymphoid cells at antiviral gene loci, along with elevated cytokine responses to unrelated stimuli, hallmarks of trained immunity. These findings suggest that modern vaccine platforms, including mRNA and viral vectors, may unintentionally induce TRIM-like responses that extend beyond antigen-specific immunity. The consequences of that are currently unclear.

Although TRIM induced by vaccines is broadly protective, its efficacy is context dependent and varies by pathogen. A meta-analysis of clinical trials showed that BCG vaccination reduced the risk of some nontuberculosis respiratory infections, such as influenza and RSV, by approximately 44% but was far less effective against SARS-CoV-2 (148, 152). This selectivity is likely shaped by the distinct immunological features of each infection, highlighting the context-dependent outcomes of trained immunity. For example, SARS-CoV-2 has evolved mechanisms to suppress innate immunity and evade interferon-mediated defenses (153), a strategy shared by many viruses (154). While TRIM may shift this balance in favor of the host, its effectiveness cannot be assumed and must be evaluated in pathogen-specific contexts through clinical studies.

To enhance vaccine-induced TRIM, one approach is to encapsulate immunogens within EVs or nanoparticles. Such packaging may allow for targeted delivery to key TRIM-relevant cells, such as macrophages or myeloid progenitors, improving both safety and efficacy. Targeting progenitors in particular may favor trained immunity over trained tolerance (155). However, the immunological consequences of packaging are complex: for example, EVs derived from *Bacteroides thetaiotaomicron*, a gut commensal, have been shown to induce trained tolerance rather than immunity (156). Although this approach remains to be experimentally validated, it may allow greater control over vaccine dosage and dosing intervals — potentially reducing the likelihood of maladaptive TRIM responses.

## Conclusions and outstanding questions

**Figure 2:**
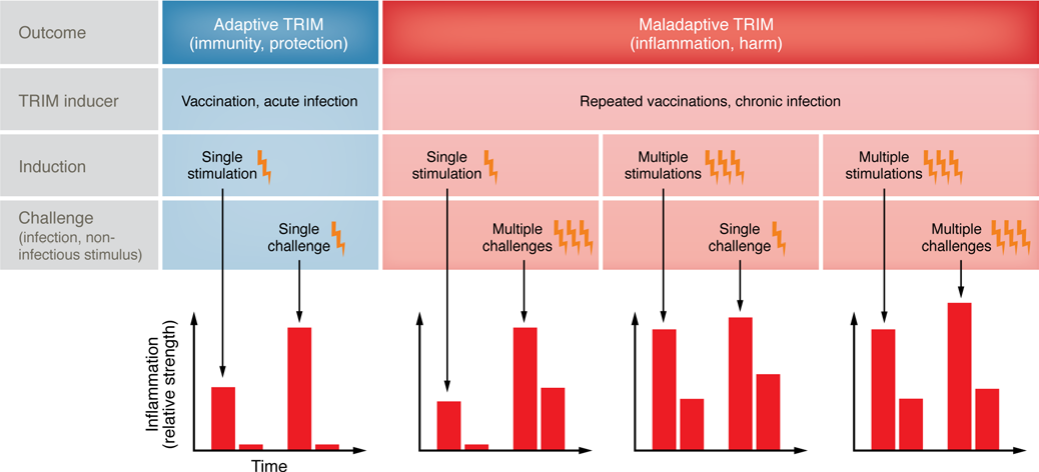
Balancing TRIM responses: context-dependent outcomes. TRIM can result in either protective or maladaptive outcomes, depending on the context and duration of stimulation. Following exposure to infectious or endogenous stimuli, innate immune cells undergo epigenetic and metabolic reprogramming, leading to an initial inflammatory response. In the case of a single, transient exposure, such as vaccination or acute infection, this response typically resolves, resulting in protective TRIM. However, repeated or prolonged stimulation, as seen with chronic infections or frequent immunizations, may drive maladaptive TRIM characterized by sustained inflammation. The nature of the secondary stimulus also influences the outcome: successful resolution of infection supports adaptive responses, while persistent infection or excessive inflammatory signaling favors maladaptive TRIM. Understanding these dynamics is critical for optimizing vaccine strategies and managing chronic immune activation.

The balance between protective and maladaptive forms of TRIM is predominantly shaped not by specific intrinsic differences between the stimuli, but by contextual and quantitative factors such as the duration, frequency, and intensity of exposure, as well as the underlying inflammatory environment (Figure 2). Ultimately, TRIM can be viewed not as a fixed outcome, but rather as a spectrum of functional states shaped by context. TRIM is, essentially, an enhanced capacity of innate cells to respond to secondary stimuli. Whether this heightened reactivity results in protective or pathological consequences depends on the interaction among the nature of the inducing signal; its duration and frequency; the underlying inflammatory status before, during and between the two stimulations; and the capacity of the system to appropriately regulate or terminate the response, reflecting an imbalance between activation and resolution of inflammation. This context dependence is especially relevant in viral infections, where acute, self-limited exposures may induce beneficial TRIM that enhances pathogen clearance, while persistent or repetitive stimulation, as seen in HIV, HBV, or latent herpesvirus infections, can drive maladaptive TRIM through epigenetic and metabolic reprogramming. This maladaptive state is marked by chronic inflammation, immune exhaustion, and worsened outcomes in settings such as secondary bacterial infections or sepsis. Understanding TRIM as a dynamic and potentially reversible process is therefore critical in chronic viral disease.

Redirecting maladaptive TRIM toward a protective phenotype represents a promising therapeutic objective. Although translational efforts are still emerging, preclinical studies suggest that targeting key TRIM-associated metabolic and epigenetic pathways, such as glycolysis, oxidative phosphorylation, and cholesterol biosynthesis, may help restore immune balance. Inhibitors of mTOR, HIF-1α, and histone methyltransferases have shown efficacy in animal models by dampening inflammation triggered by viral components (157). Additionally, interventions at the level of hematopoietic progenitors, including metabolic modulation and epigenetic remodeling, may allow reprogramming of innate immunity to prevent postviral complications (158).

Key outstanding questions include: (i) Which viral infections are most likely to induce maladaptive TRIM, and by what mechanisms? (ii) Can a composite quantitative measure be calculated that reflects a probability of TRIM becoming maladaptive? (iii) What epigenetic and metabolic features differentiate adaptive from maladaptive TRIM in myeloid and nonmyeloid cells? (iv) Can these features be leveraged to guide therapeutic reprogramming or risk stratification? (v) Are virus-induced TRIM effects reversible following viral clearance or antiretroviral therapy in HIV? Signatures based on chromatin accessibility, histone modifications, transcriptional profiles, and metabolic flux may serve both diagnostic and interventional roles, identifying individuals at risk for postviral complications and informing the use of therapies aimed at reprogramming maladaptive TRIM. This dual utility positions TRIM signatures as a powerful translational tool.

Ultimately, deepening our understanding of how viruses modulate TRIM will uncover new strategies to counteract virus-induced chronic inflammation, sepsis susceptibility, and immune dysfunction. Therapeutic manipulation of TRIM offers an innovative avenue to restore immune homeostasis and reduce the long-term consequences of both acute and persistent viral infections. Moreover, insights gained from viral contexts may extend to noninfectious diseases characterized by dysregulated TRIM.

## Figures and Tables

**Table 1:**
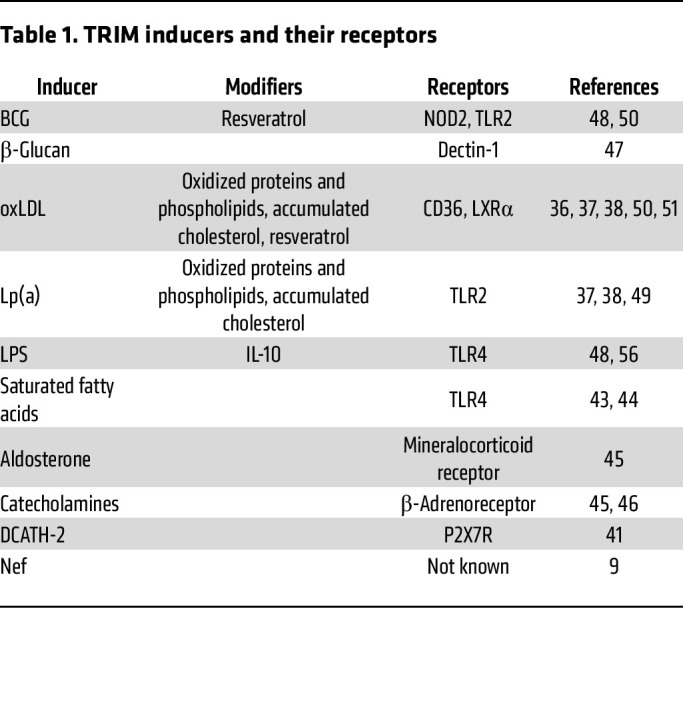
TRIM inducers and their receptors

## Version 1 09/02/2025

Electronic publication
